# Supplementary material for: IL-33-Induced Transcriptional Activation of LPIN1 Accelerates Breast Tumorigenesis
Source: Cancers (Basel). 2021 Apr 30;13(9):2174. doi: 10.3390/cancers13092174 (PMC8124251; doi:10.3390/cancers13092174)

Figure1. A

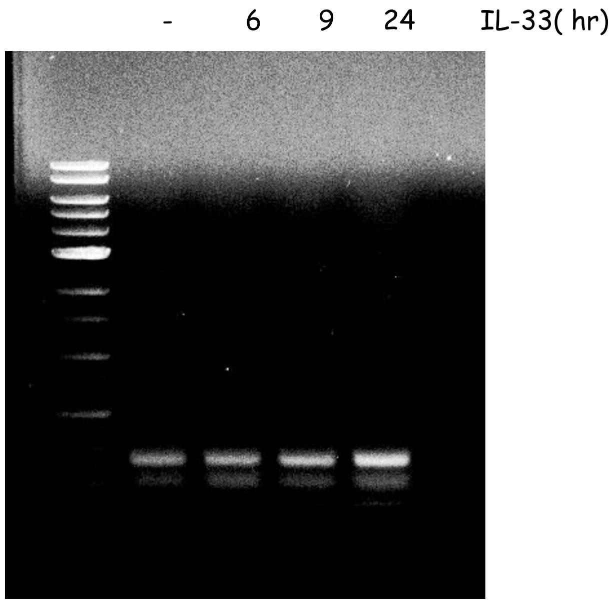

LPIN1

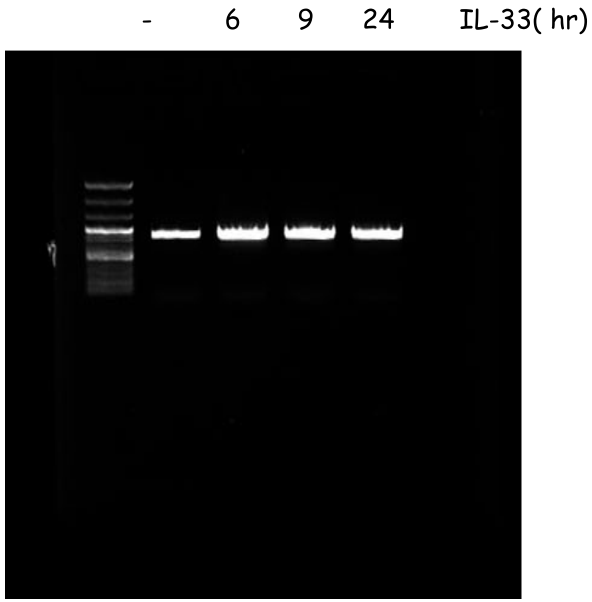

β-actin

Figure1. C

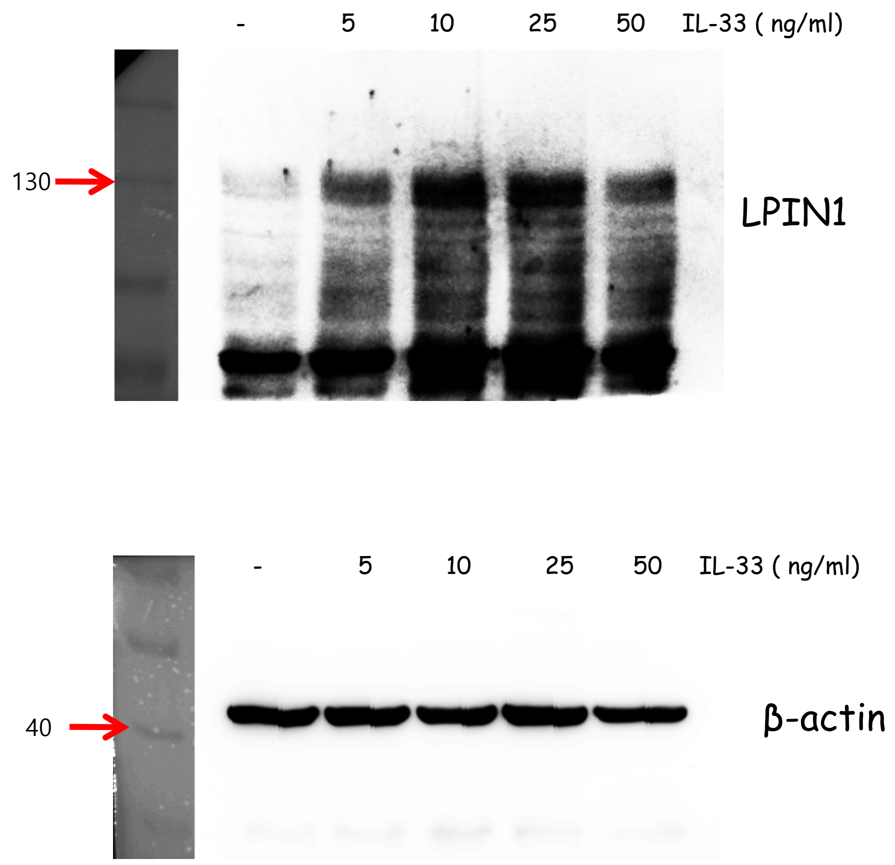

Figure1. D

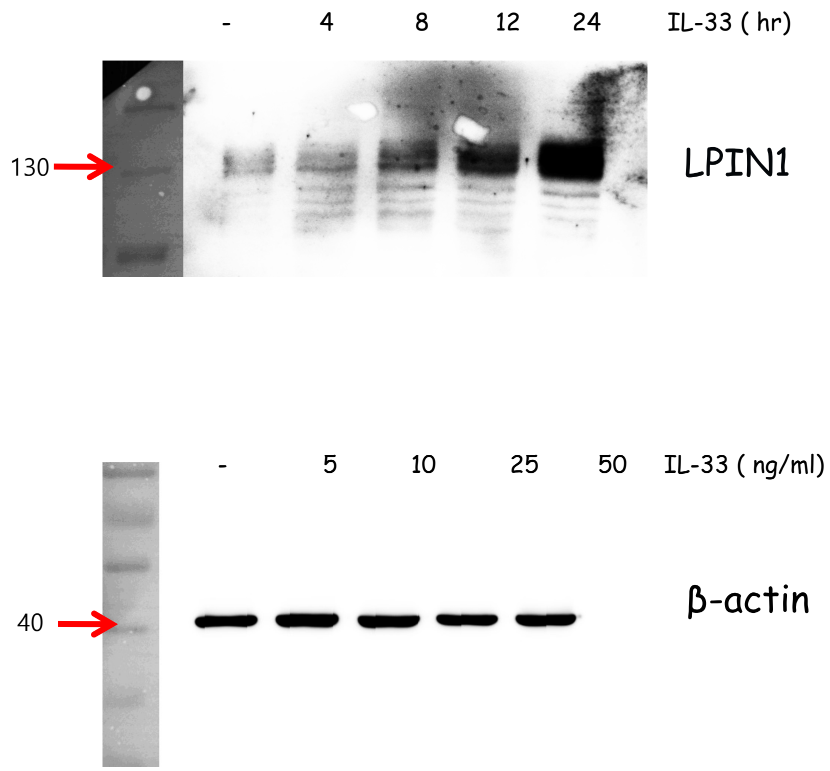

Figure1. E

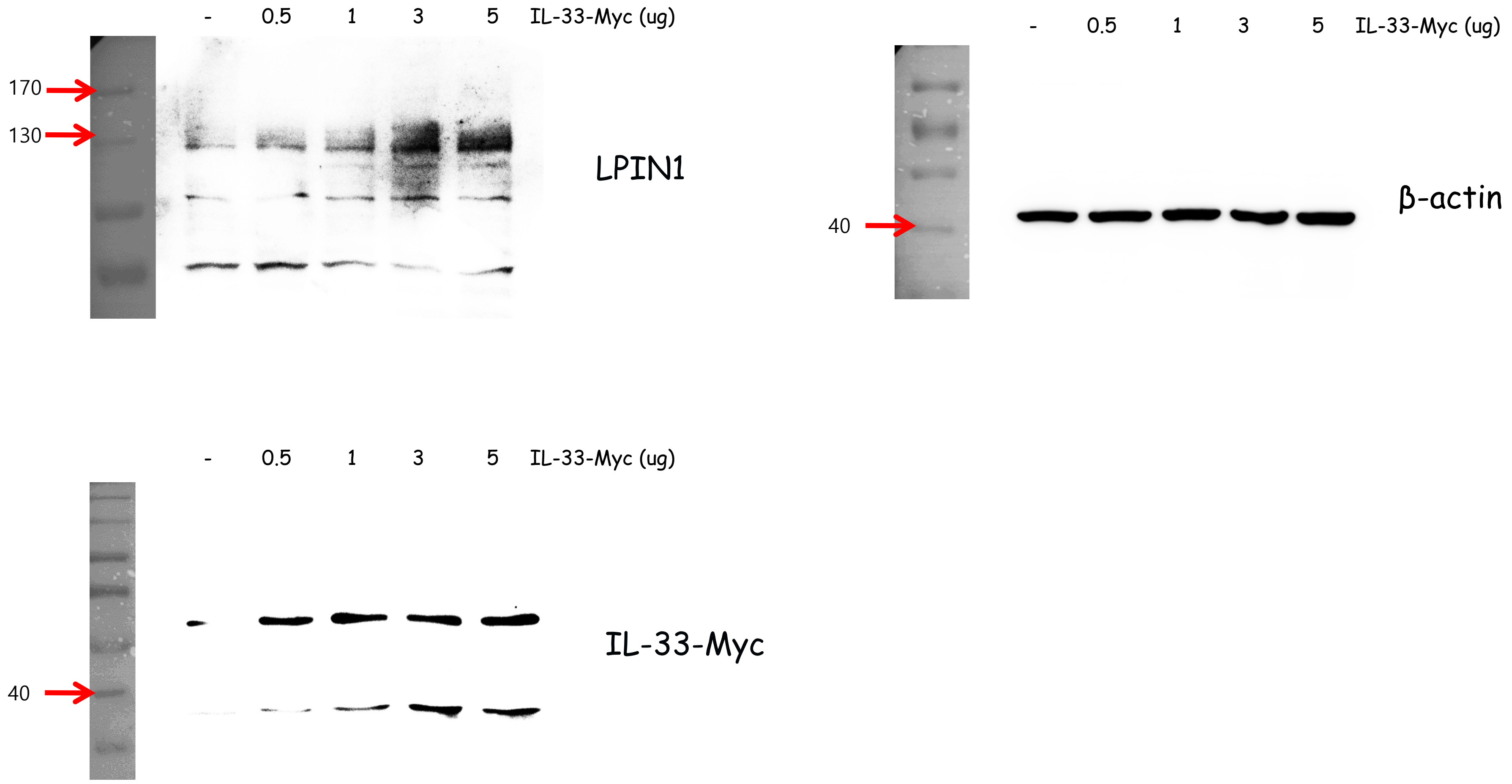

- 0.5 1 3 5 IL-33-Myc (ug)
170
130
LPIN1
- 0.5 1 3 5 IL-33-Myc (ug)
40
β-actin
- 0.5 1 3 5 IL-33-Myc (ug)
40
IL-33-Myc

Figure1. F

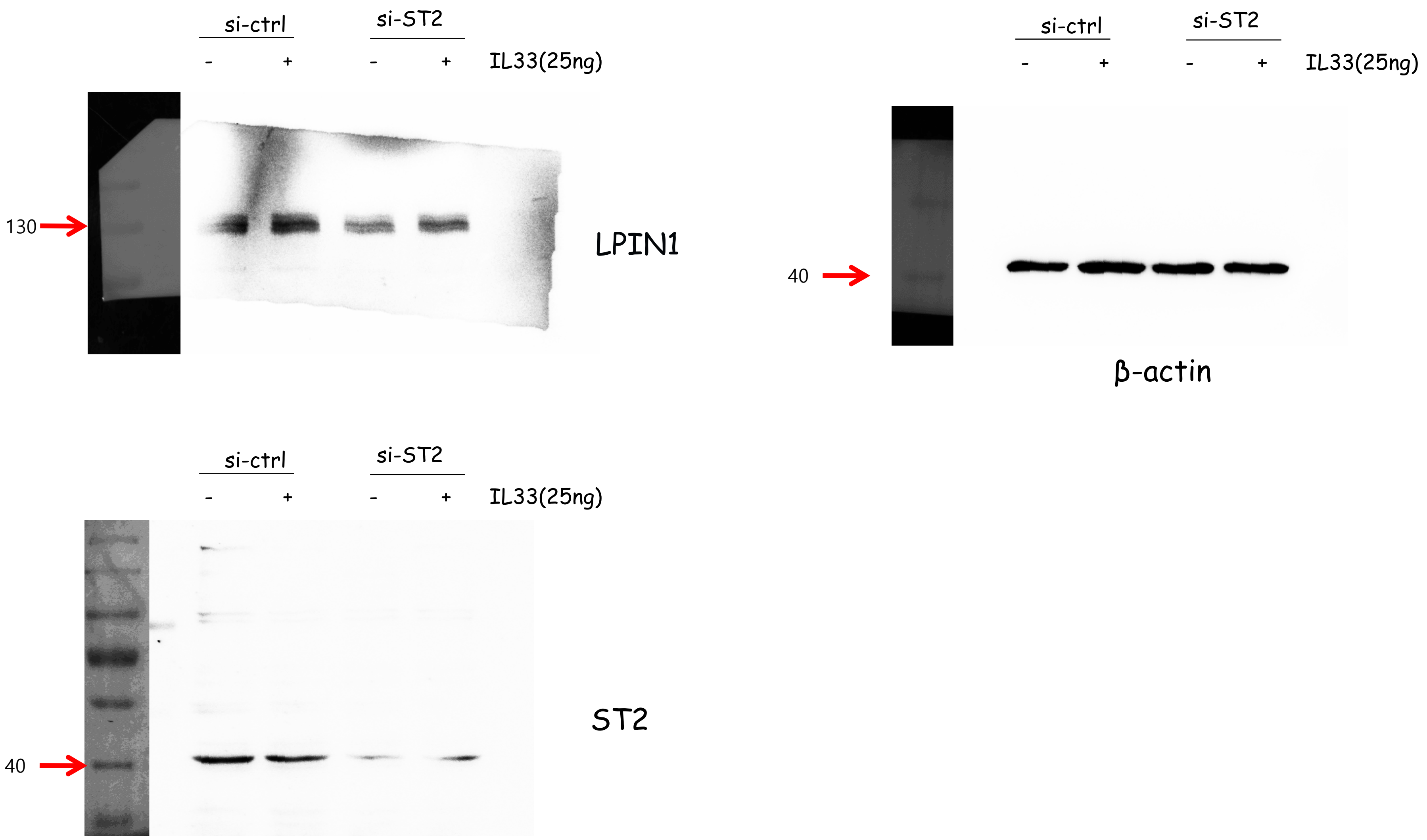
si-ctrl
si-ST2
- + - + IL33(25ng)
130
LPIN1
si-ctrl
si-ST2
- + - + IL33(25ng)
40
β-actin
si-ctrl
si-ST2
- + - + IL33(25ng)
40
ST2

Figure2.A

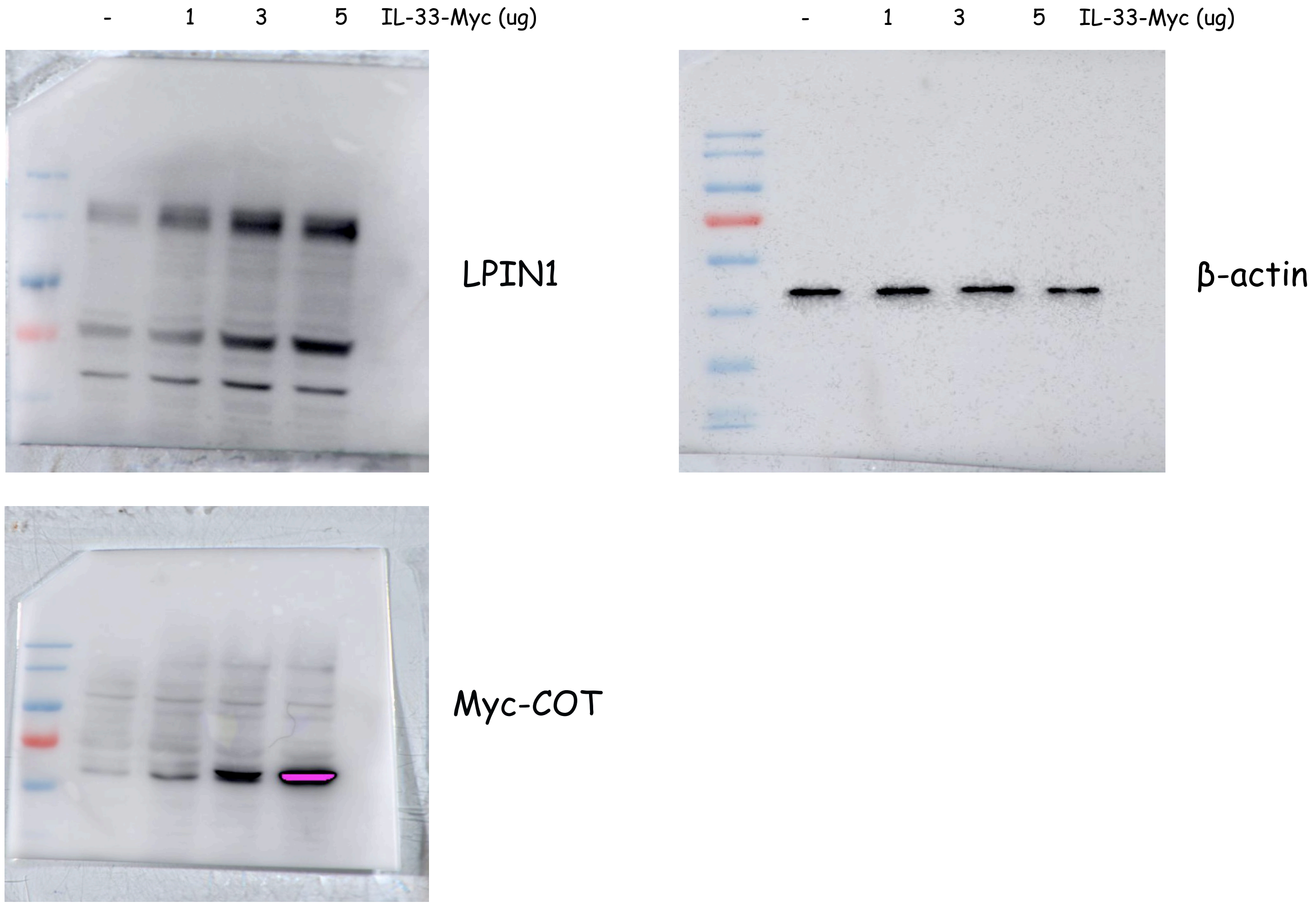

Figure2.B

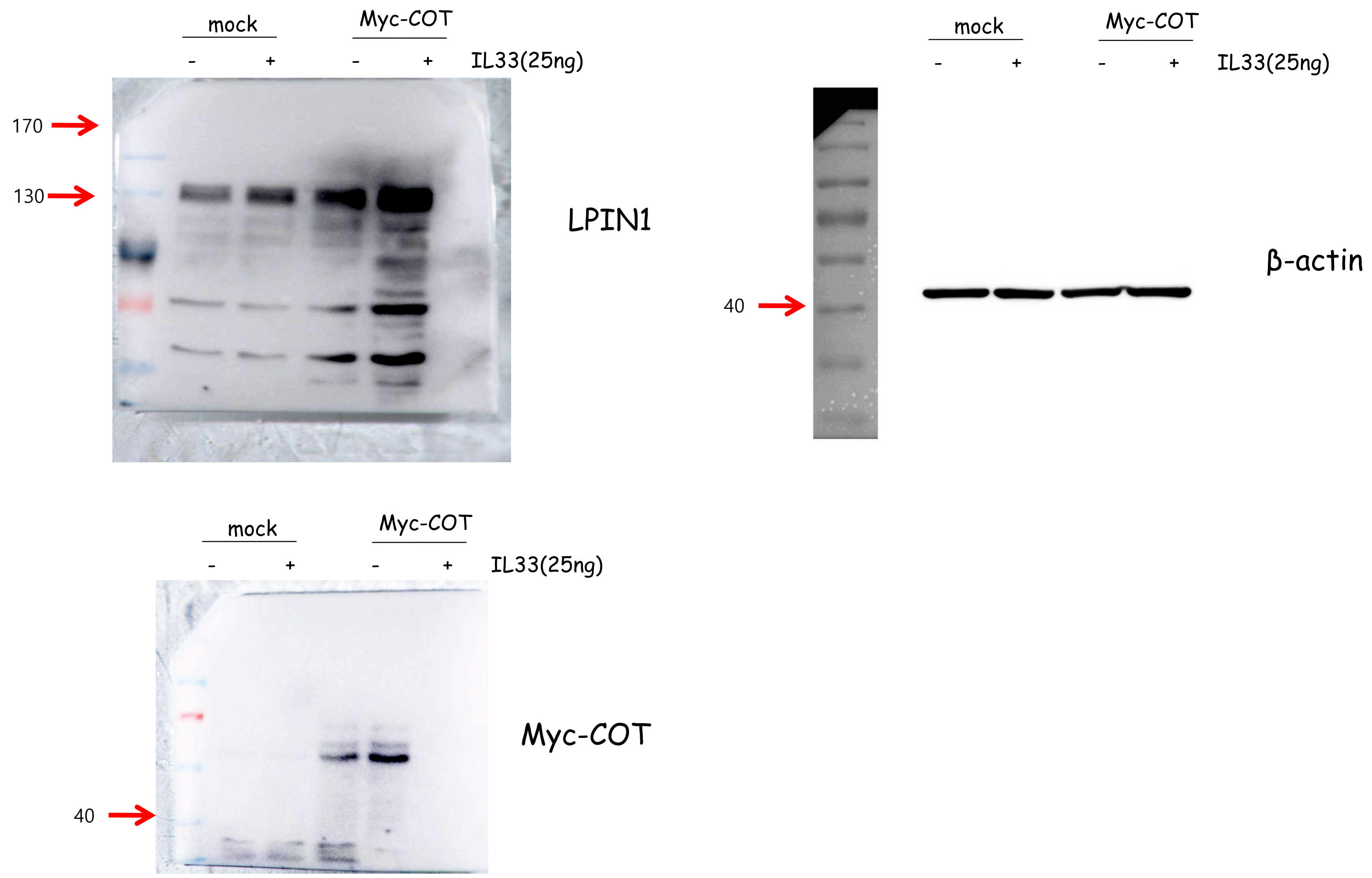
mock
Myc-COT
- + - + IL33(25ng)
170
130
LPIN1
mock
Myc-COT
- + - + IL33(25ng)
40
β-actin
mock
Myc-COT
- + - + IL33(25ng)
40
Myc-COT

Figure2.C

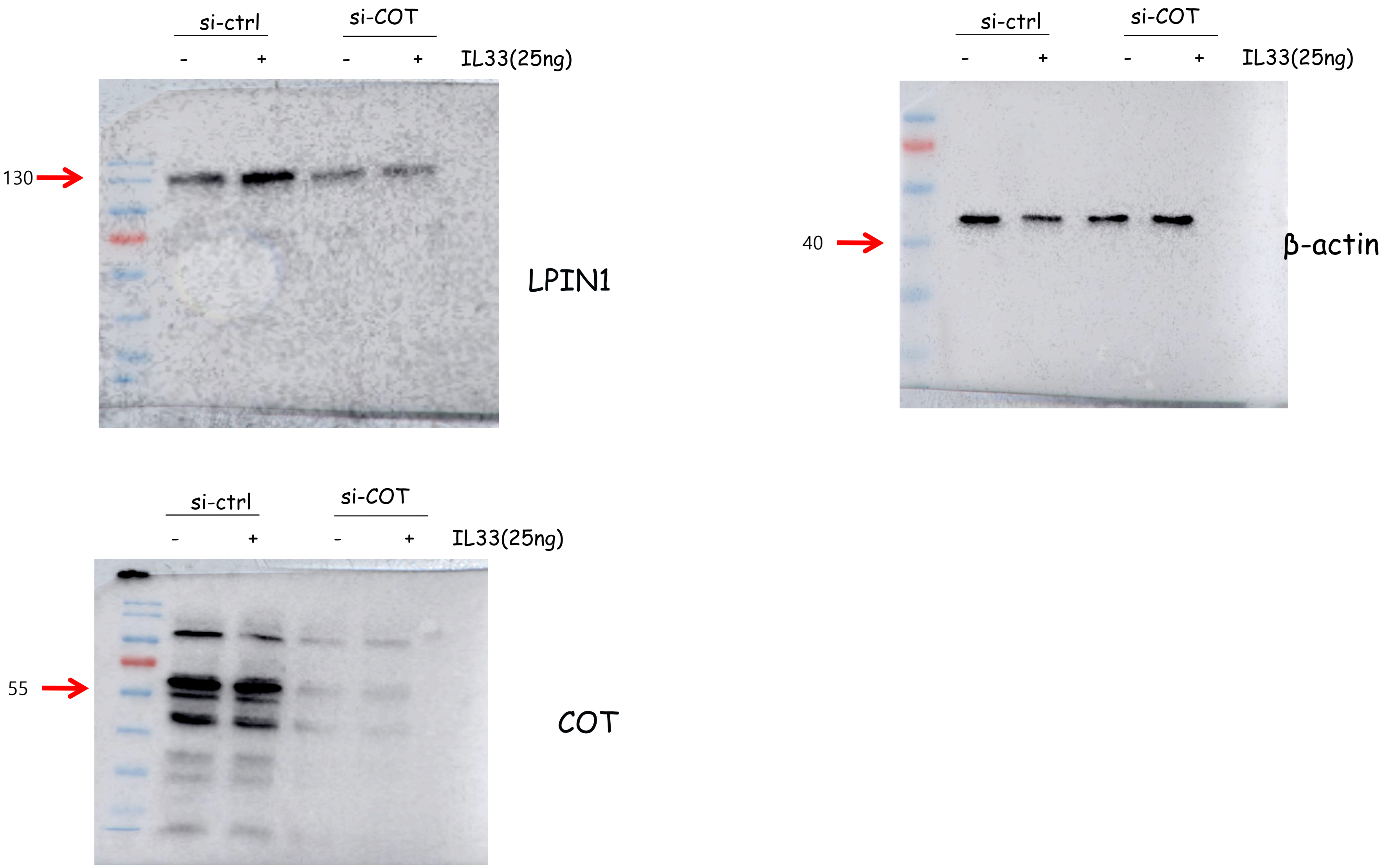
si-ctrl
si-COT
- + - + IL33(25ng)
130
LPIN1
si-ctrl
si-COT
- + - + IL33(25ng)
40
β-actin
si-ctrl
si-COT
- + - + IL33(25ng)
55
COT

Figure2.D

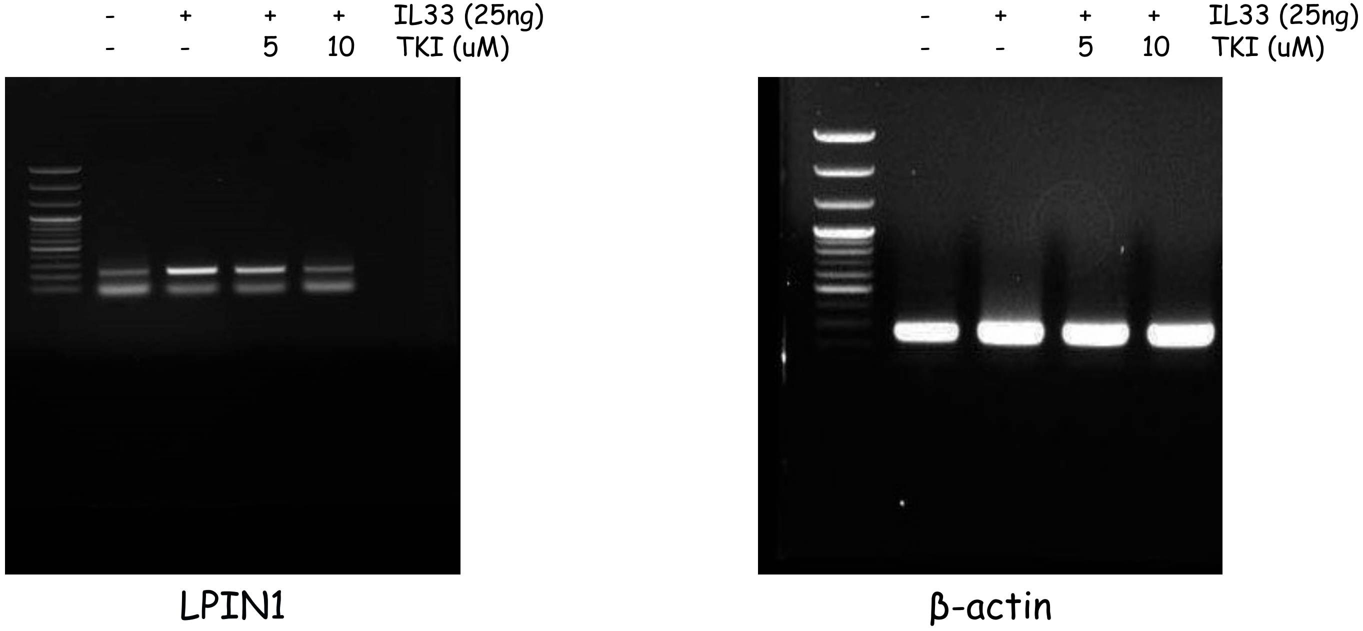

Figure2. F

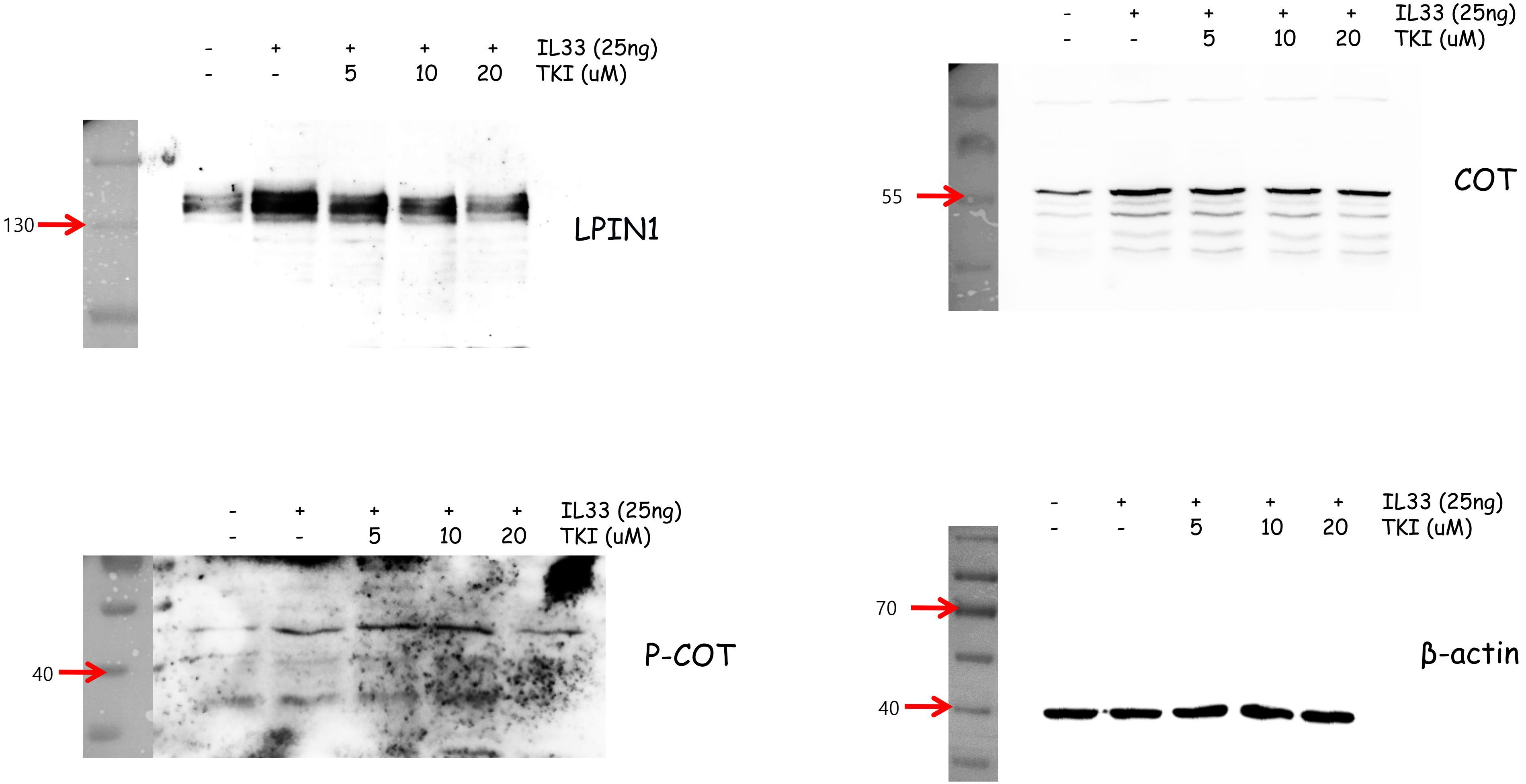
- + + + + IL33 (25ng)
- - 5 10 20 TKI (uM)
130
LPIN1
- + + + + IL33 (25ng)
- - 5 10 20 TKI (uM)
55
COT
- + + + + IL33 (25ng)
- - 5 10 20 TKI (uM)
40
P-COT
- + + + + IL33 (25ng)
- - 5 10 20 TKI (uM)
70
40
β-actin

Figure3. A

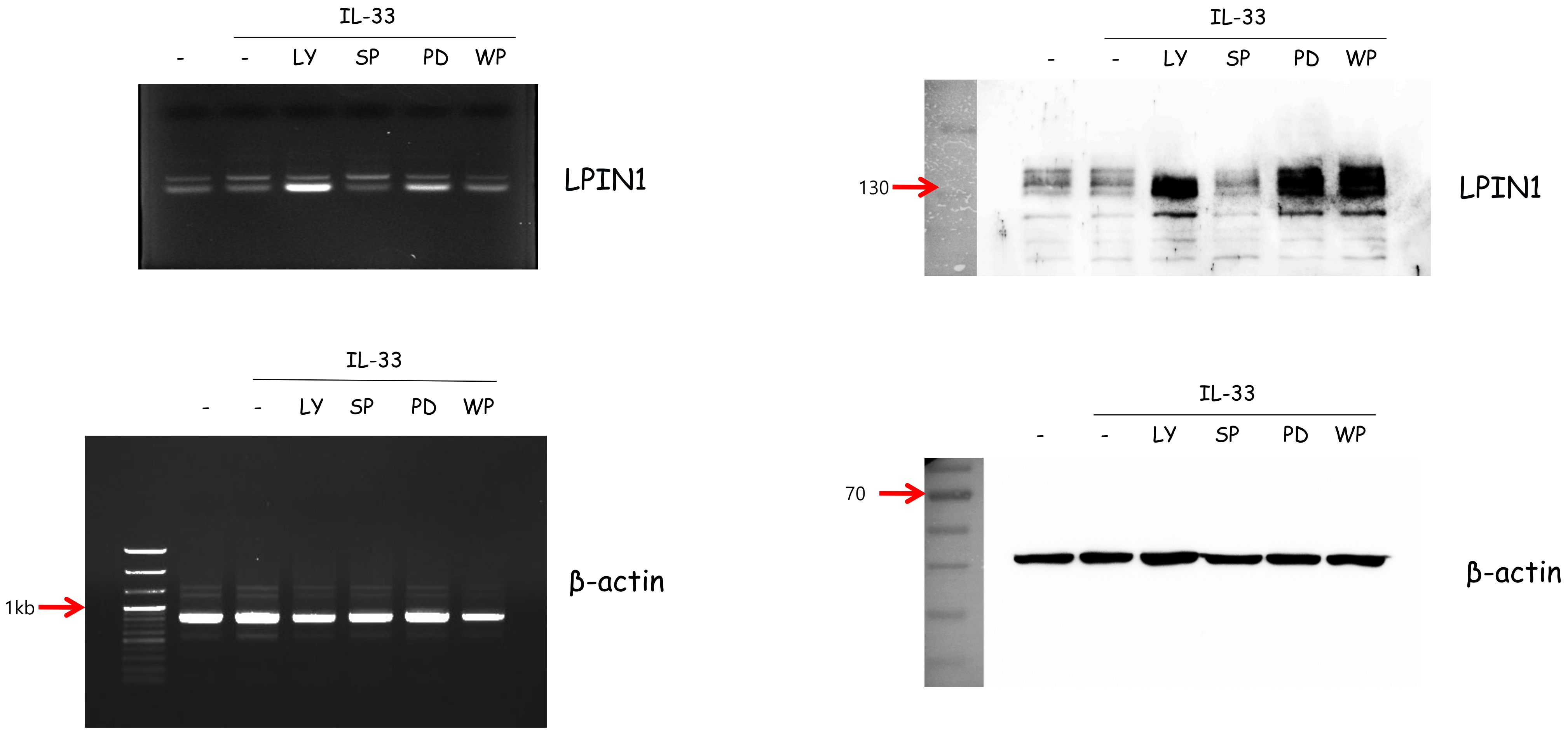

IL-33
- - LY SP PD WP
LPIN1
IL-33
- - LY SP PD WP
130
LPIN1
IL-33
- - LY SP PD WP
1kb
β-actin
IL-33
- - LY SP PD WP
70
β-actin

Figure3. B

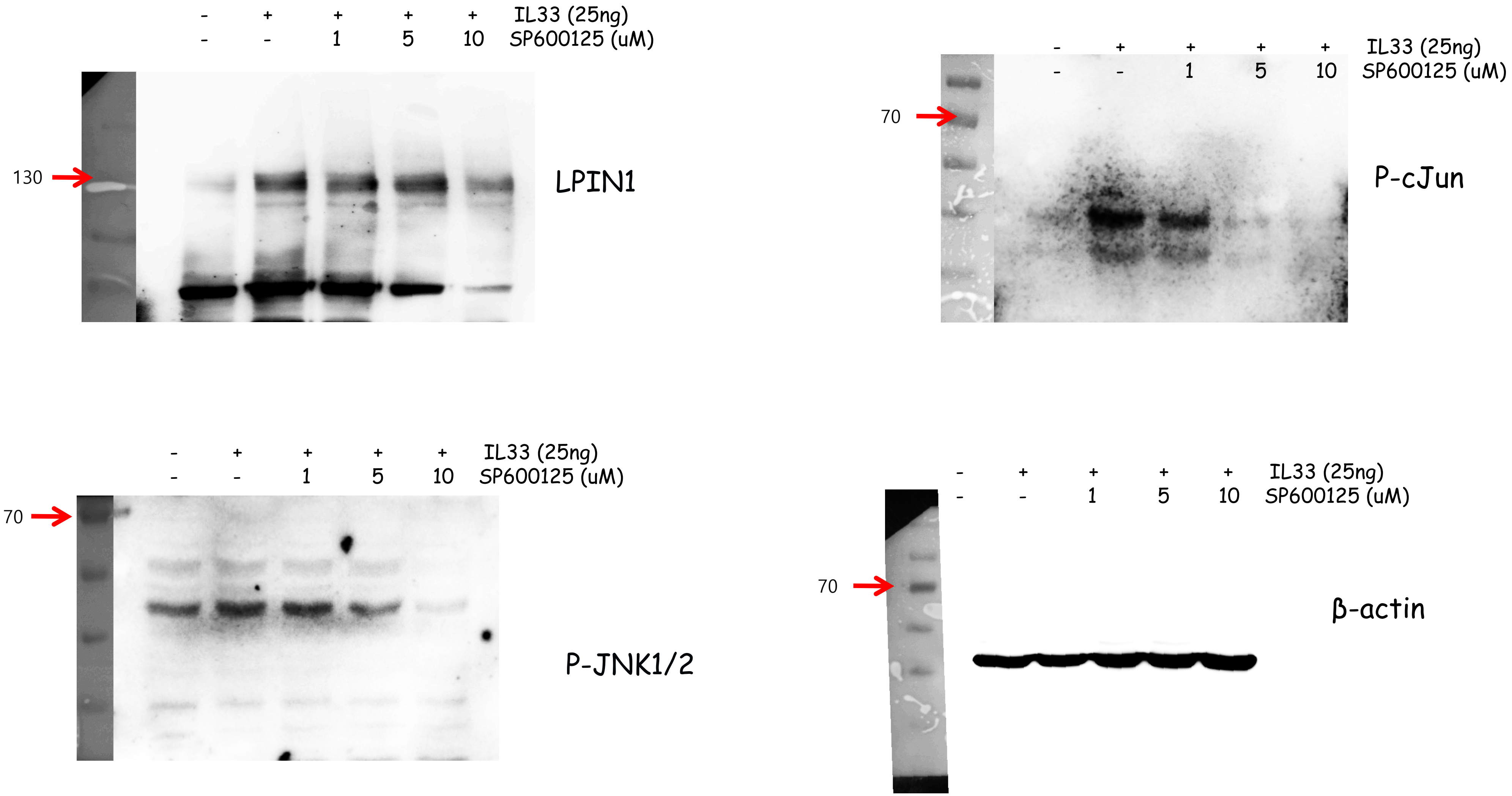
- + + + + IL33 (25ng)
- - 1 5 10 SP600125 (uM)
130
LPIN1
- + + + + IL33 (25ng)
- - 1 5 10 SP600125 (uM)
70
P-cJun
- + + + + IL33 (25ng)
- - 1 5 10 SP600125 (uM)
70
P-JNK1/2
- + + + + IL33 (25ng)
- - 1 5 10 SP600125 (uM)
70
β-actin

Figure3. C

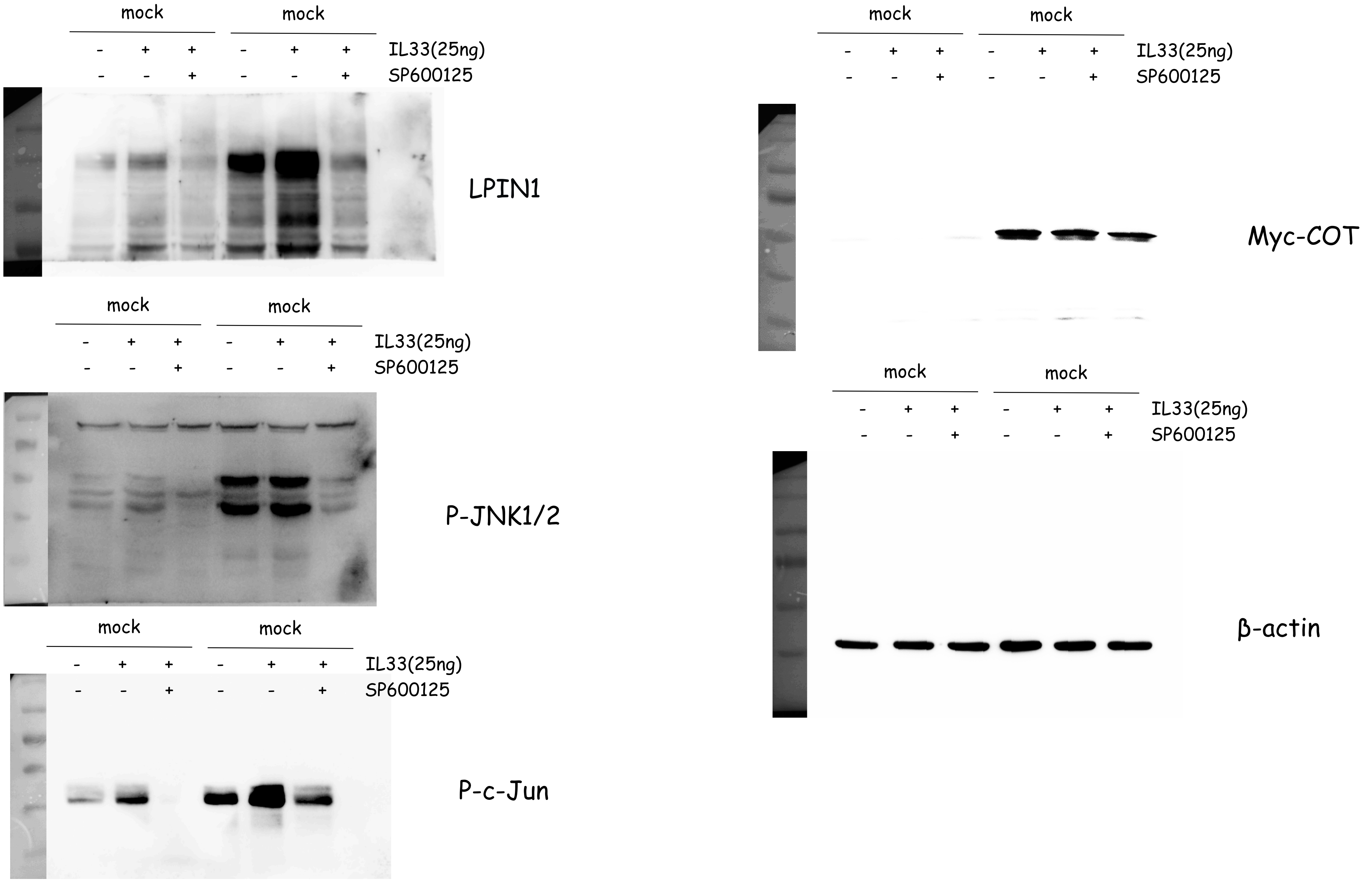
mock
mock
IL33(25ng)
SP600125
LPIN1
P-JNK1/2
P-c-Jun
Myc-COT
β-actin

Figure3. D

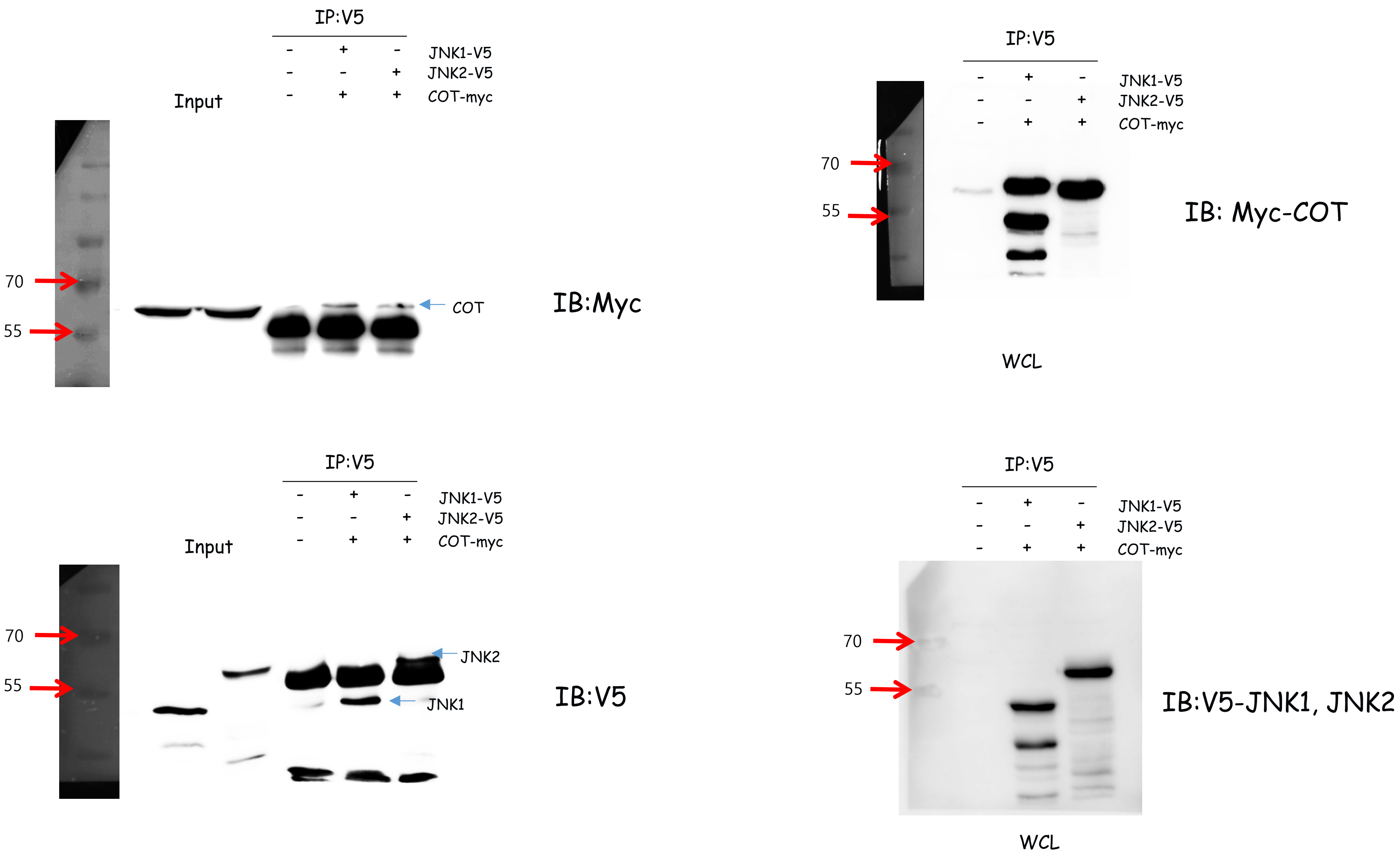
IP:V5
- + - JNK1-V5
- - + JNK2-V5
Input
- + + COT-myc
70
55
COT
IB:Myc
IP:V5
- + - JNK1-V5
- - + JNK2-V5
- + + COT-myc
70
55
IB: Myc-COT
WCL
IP:V5
- + - JNK1-V5
- - + JNK2-V5
Input
- + + COT-myc
70
55
JNK2
JNK1
IB:V5
IP:V5
- + - JNK1-V5
- - + JNK2-V5
- + + COT-myc
70
55
IB:V5-JNK1, JNK2
WCL

Figure3. E

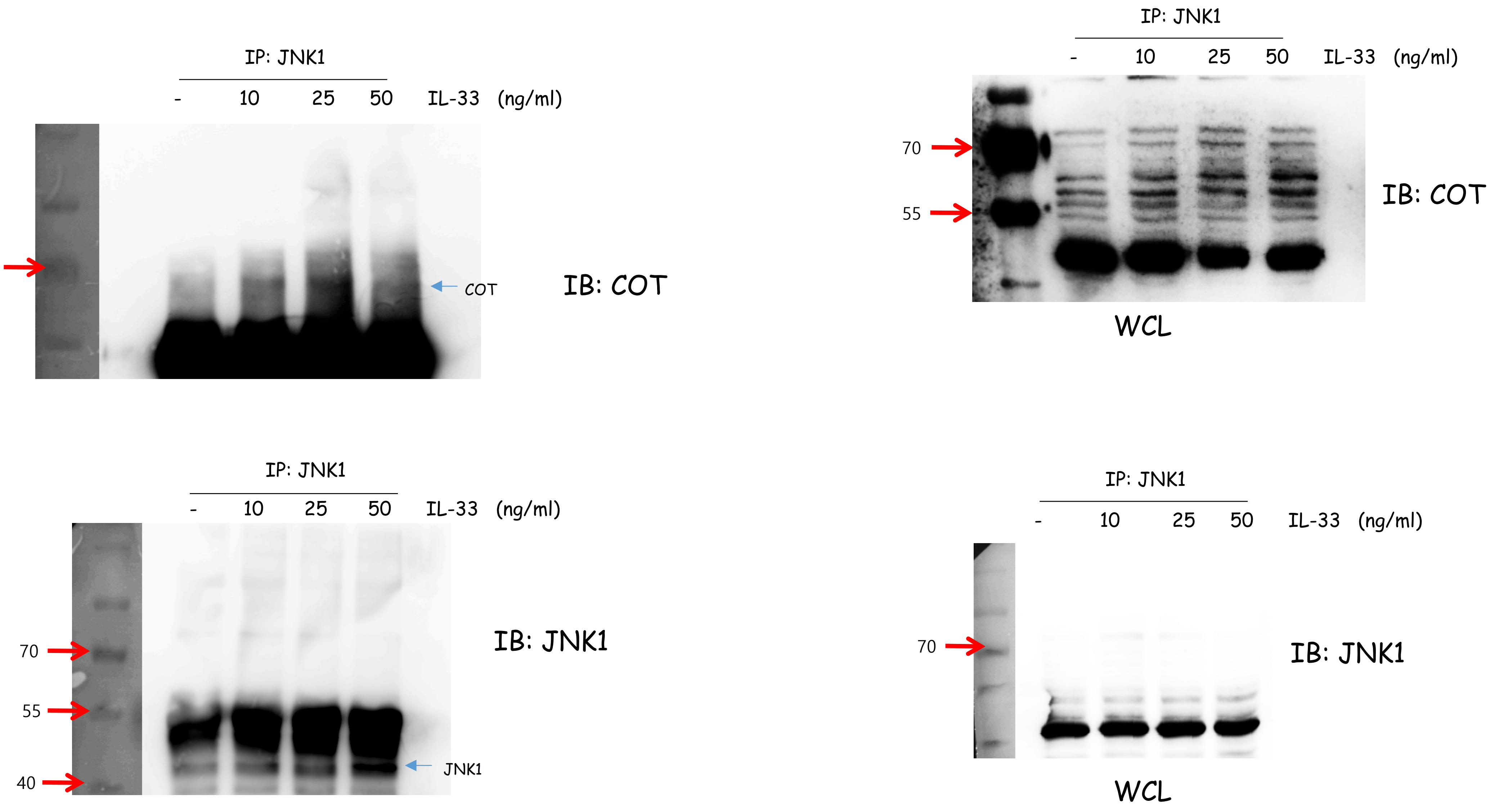
IP: JNK1
- 10 25 50 IL-33 (ng/ml)
COT
IB: COT
IP: JNK1
- 10 25 50 IL-33 (ng/ml)
70
55
IB: COT
WCL
IP: JNK1
- 10 25 50 IL-33 (ng/ml)
IB: JNK1
70
55
40
JNK1
IP: JNK1
- 10 25 50 IL-33 (ng/ml)
70
IB: JNK1
WCL

Figure3. F

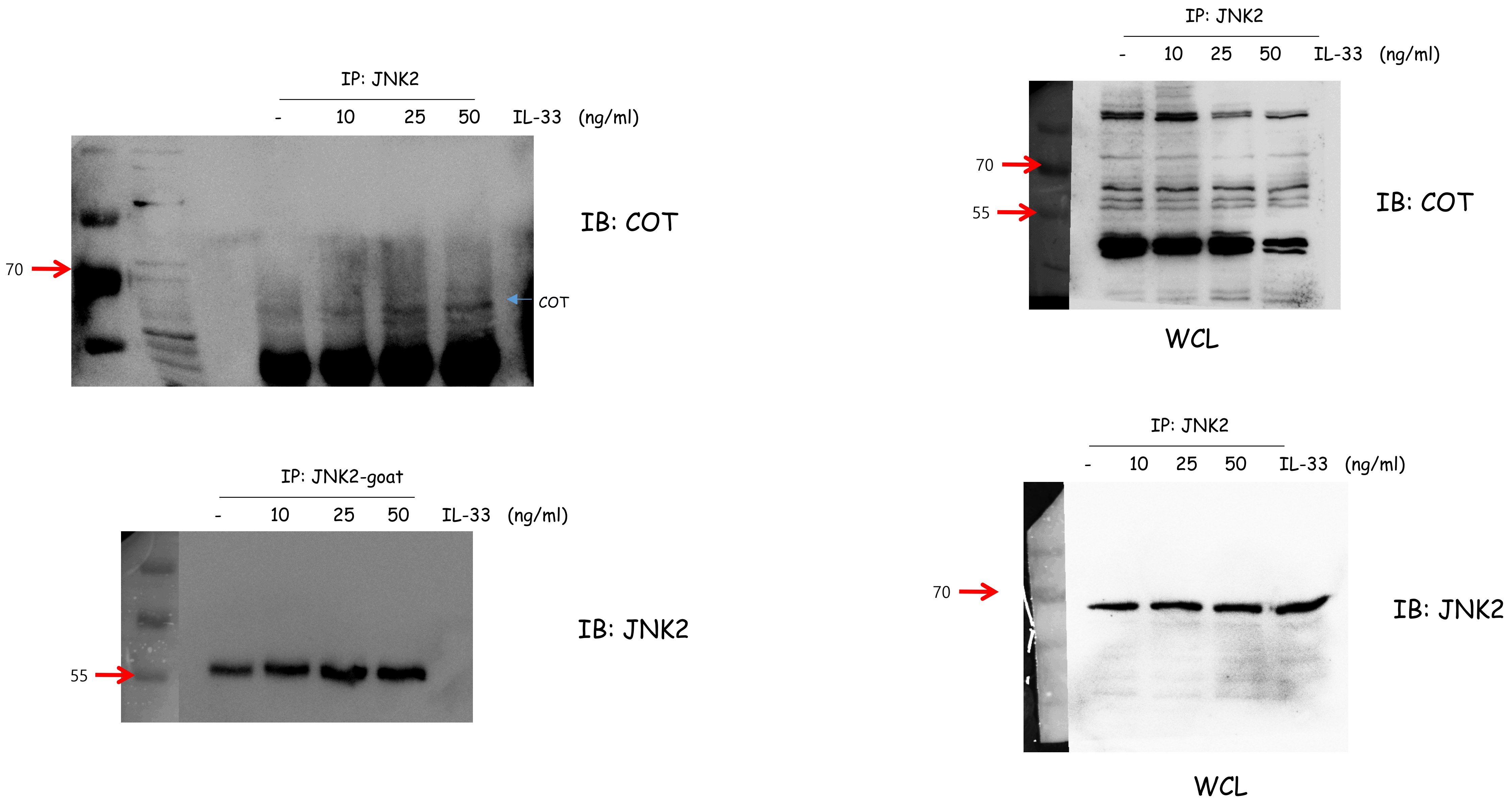
IP: JNK2
- 10 25 50 IL-33 (ng/ml)
70
COT
IB: COT
IP: JNK2
- 10 25 50 IL-33 (ng/ml)
70
55
IB: COT
WCL
IP: JNK2-goat
- 10 25 50 IL-33 (ng/ml)
55
IB: JNK2
IP: JNK2
- 10 25 50 IL-33 (ng/ml)
70
IB: JNK2
WCL

Figure3. G

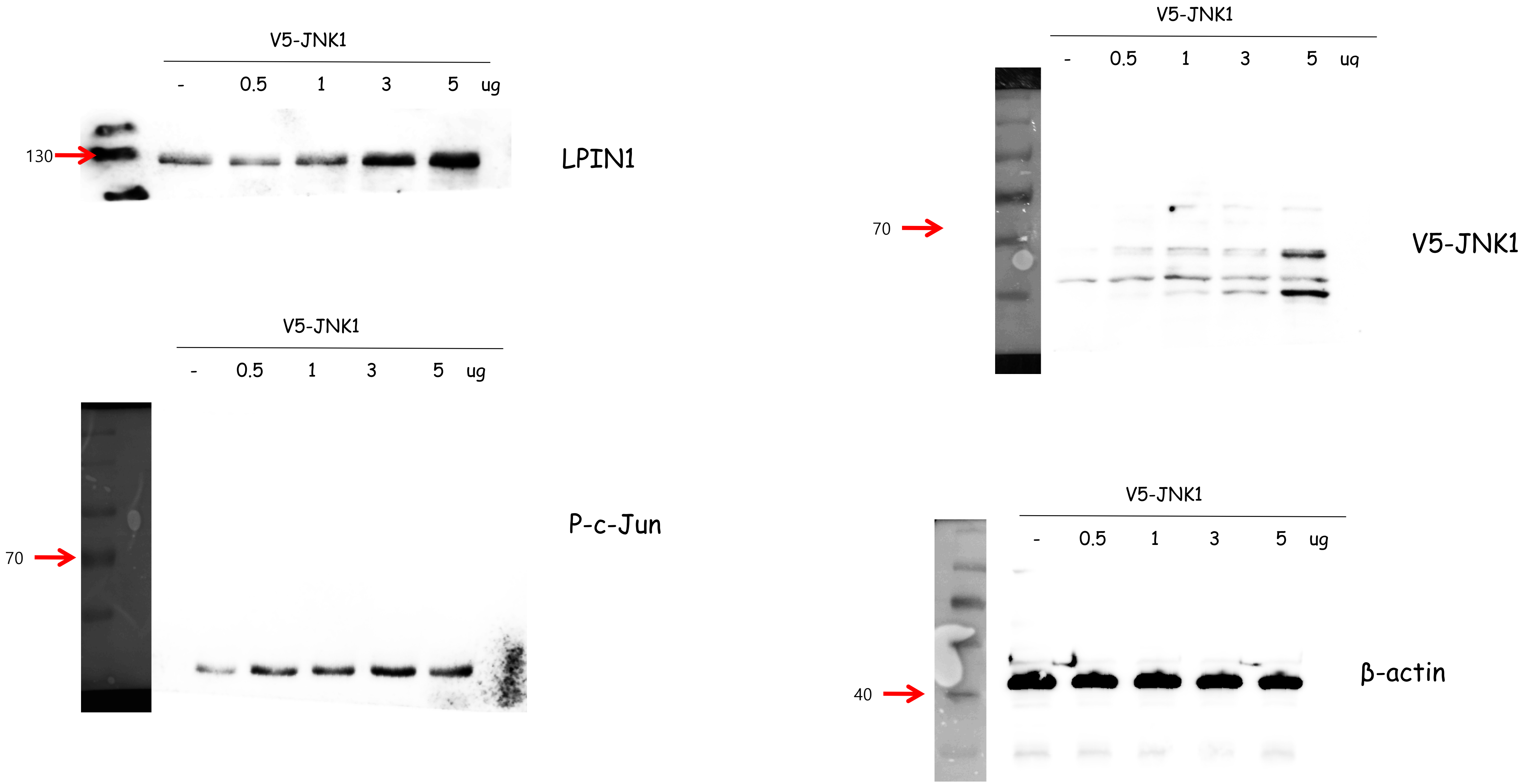
V5-JNK1
- 0.5 1 3 5 ug
130
LPIN1
V5-JNK1
- 0.5 1 3 5 ug
70
P-c-Jun
V5-JNK1
- 0.5 1 3 5 ug
70
V5-JNK1
V5-JNK1
- 0.5 1 3 5 ug
40
β-actin

Figure3. G

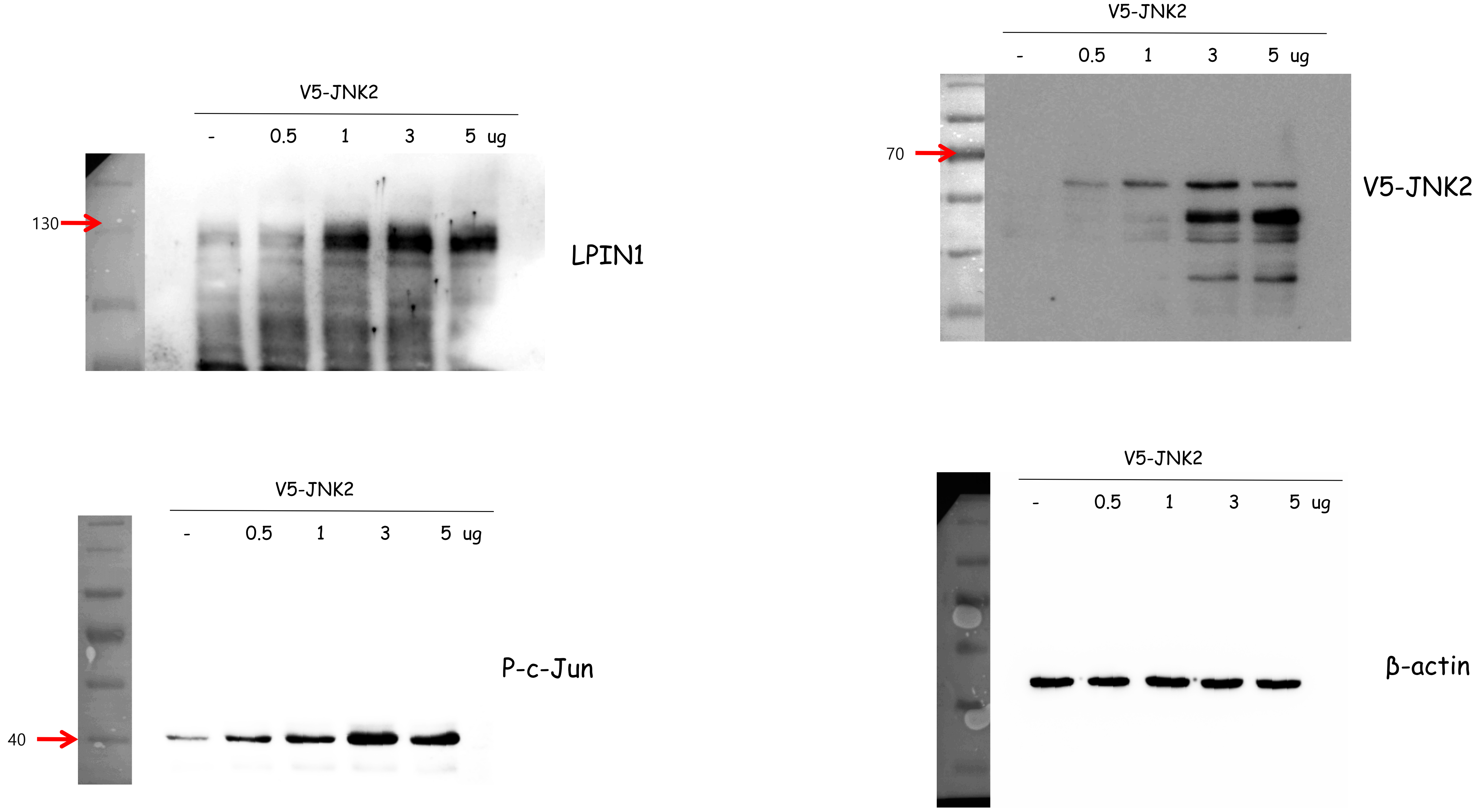

V5-JNK2
- 0.5 1 3 5 ug
130
LPIN1
V5-JNK2
- 0.5 1 3 5 ug
70
V5-JNK2
V5-JNK2
- 0.5 1 3 5 ug
40
P-c-Jun
V5-JNK2
- 0.5 1 3 5 ug
β-actin

Figure3. H

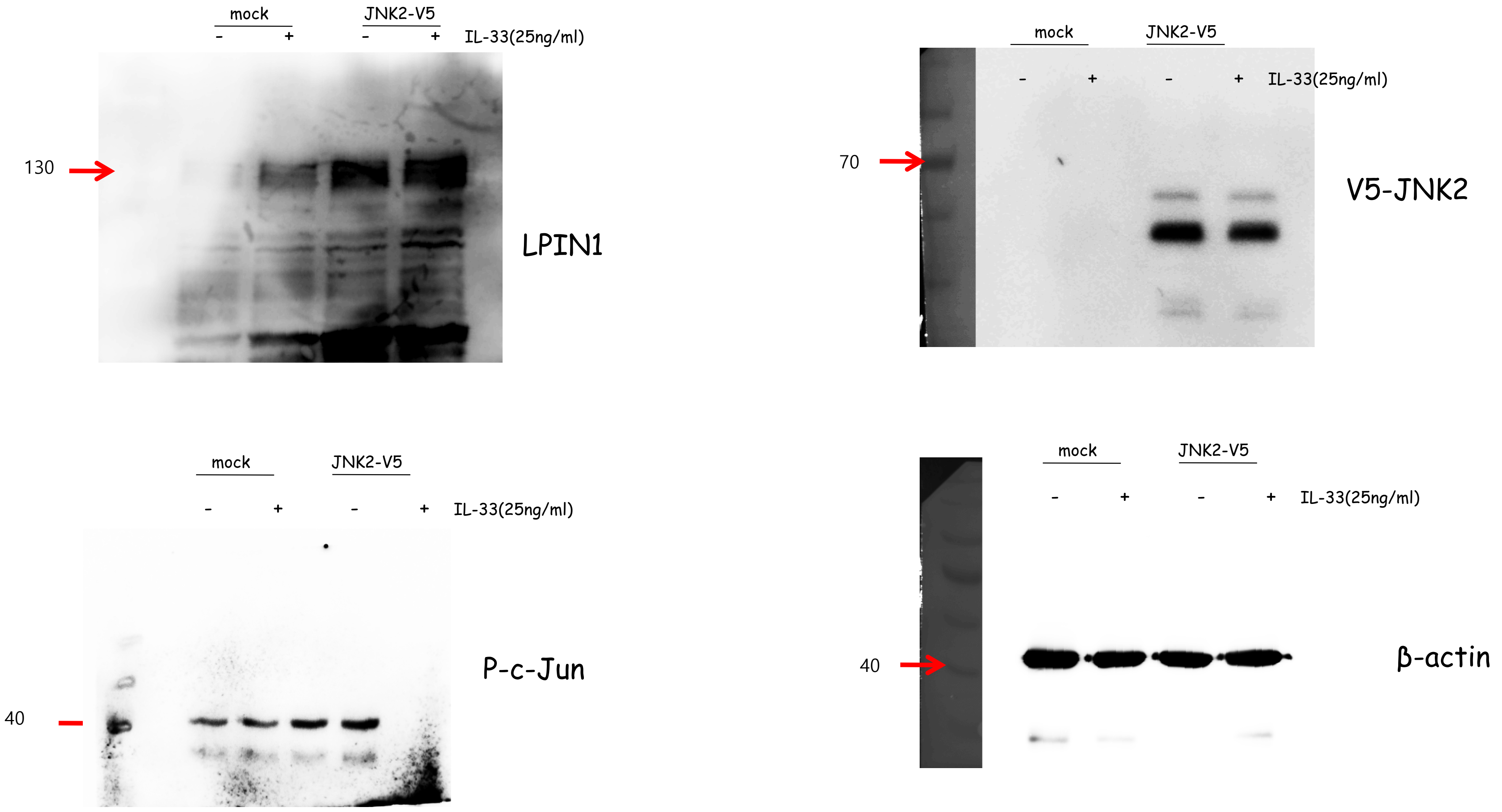
mock
JNK2-V5
- + - + IL-33(25ng/ml)
130
LPIN1
mock
JNK2-V5
- + - + IL-33(25ng/ml)
70
V5-JNK2
mock
JNK2-V5
- + - + IL-33(25ng/ml)
40
P-c-Jun
mock
JNK2-V5
- + - + IL-33(25ng/ml)
40
β-actin

Figure3. H

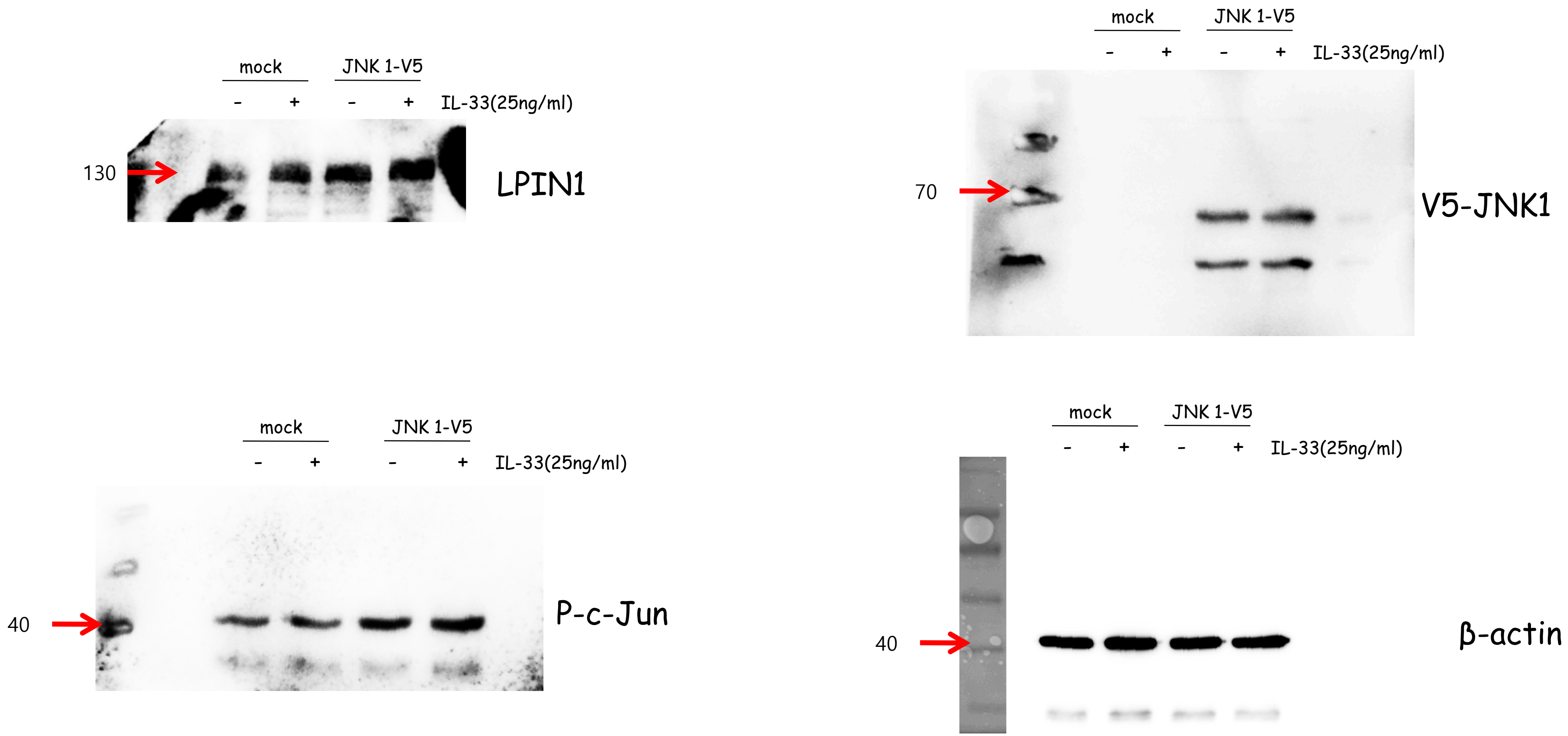

mock
JNK 1-V5
- + - + IL-33(25ng/ml)
130
LPIN1
mock
JNK 1-V5
- + - + IL-33(25ng/ml)
70
V5-JNK1
mock
JNK 1-V5
- + - + IL-33(25ng/ml)
40
P-c-Jun
mock
JNK 1-V5
- + - + IL-33(25ng/ml)
40
β-actin

Figure3. I

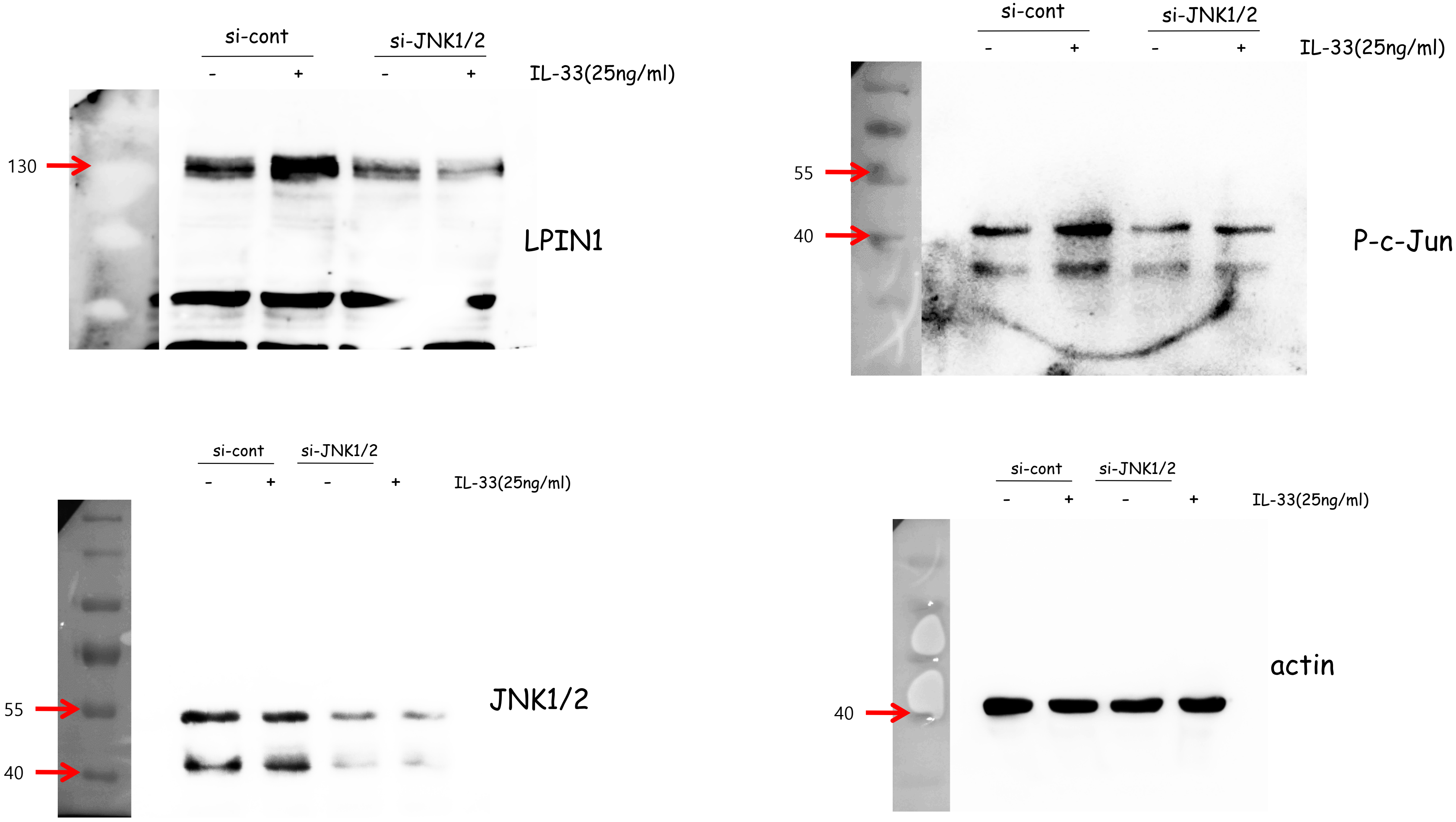

si-cont
si-JNK1/2
- + - +
IL-33(25ng/ml)
130
LPIN1
55
40
P-c-Jun
JNK1/2
actin

Figure4. A

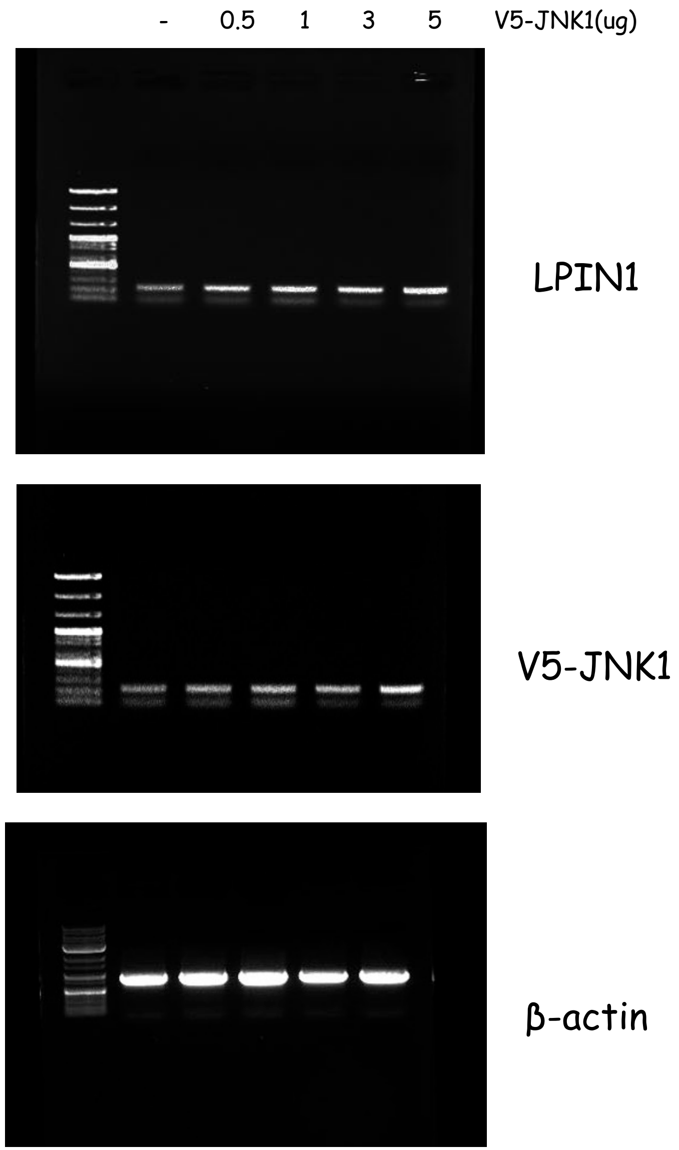

Figure4. B

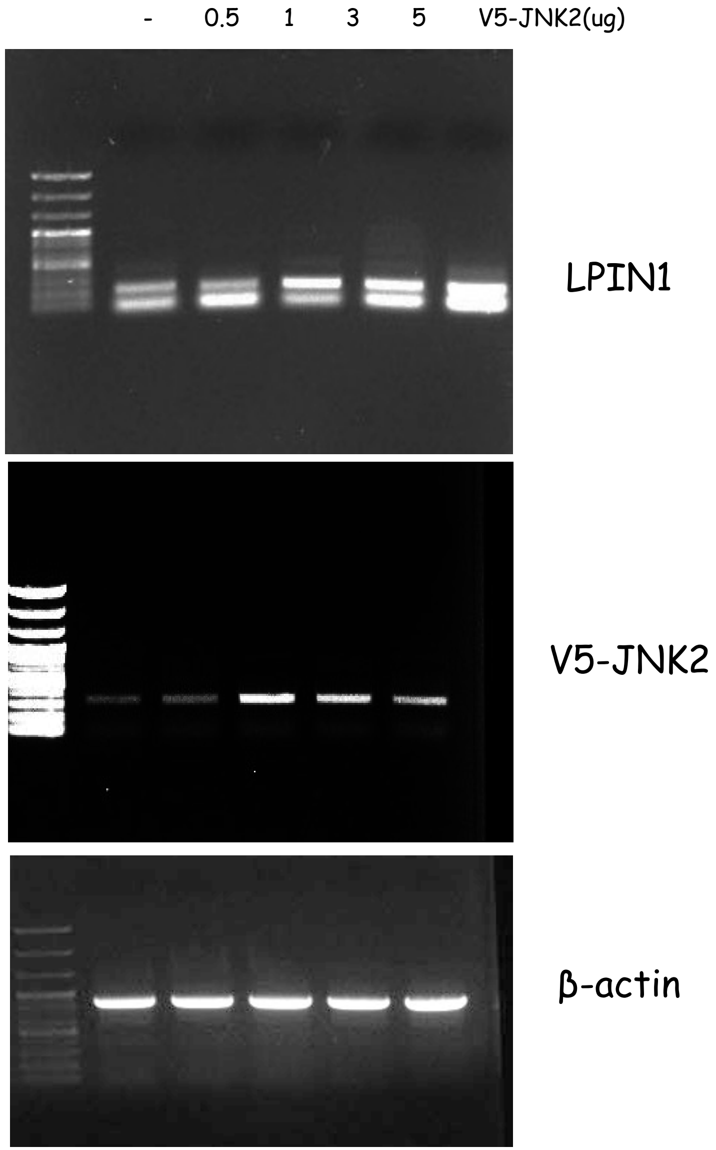

Figure4. C

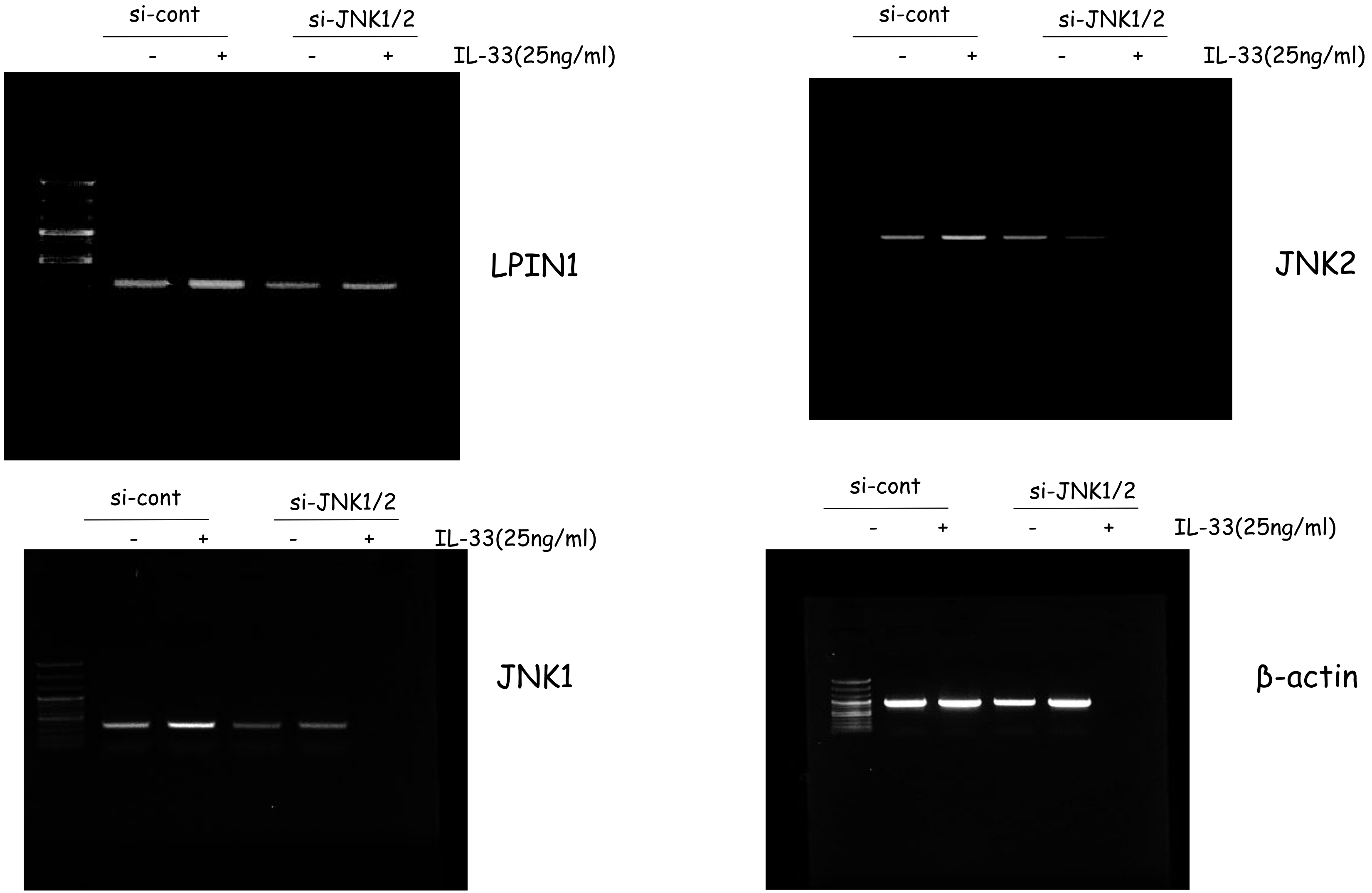

si-cont
si-JNK1/2
- + - + IL-33(25ng/ml)
LPIN1
si-cont
si-JNK1/2
- + - + IL-33(25ng/ml)
JNK2
si-cont
si-JNK1/2
- + - + IL-33(25ng/ml)
JNK1
si-cont
si-JNK1/2
- + - + IL-33(25ng/ml)
β-actin

Figure4. D

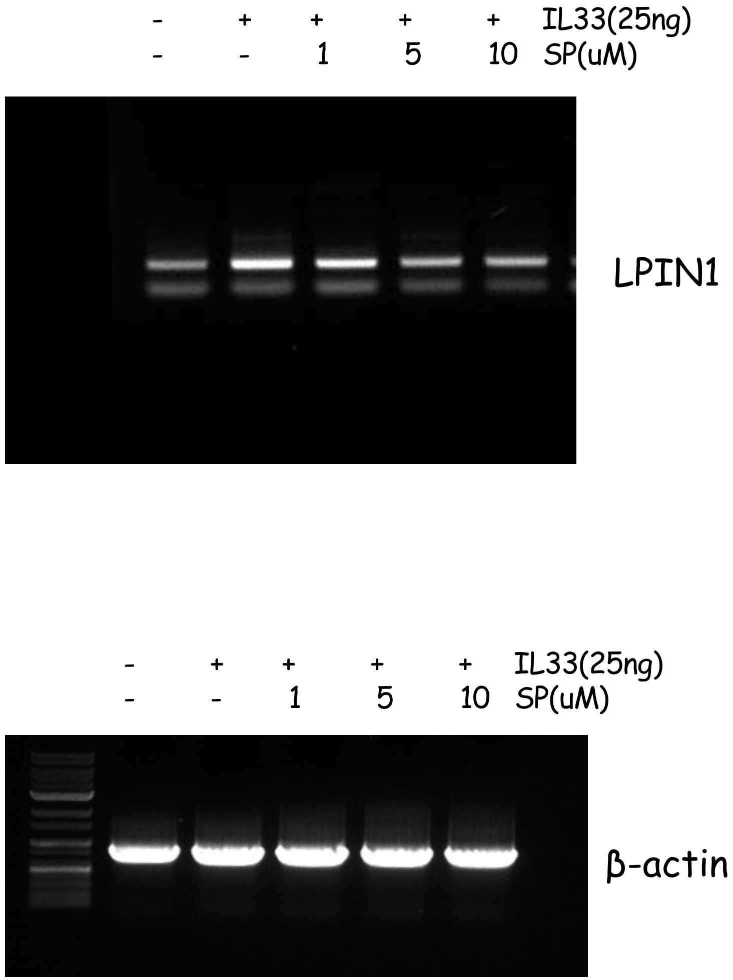

Figure4. F

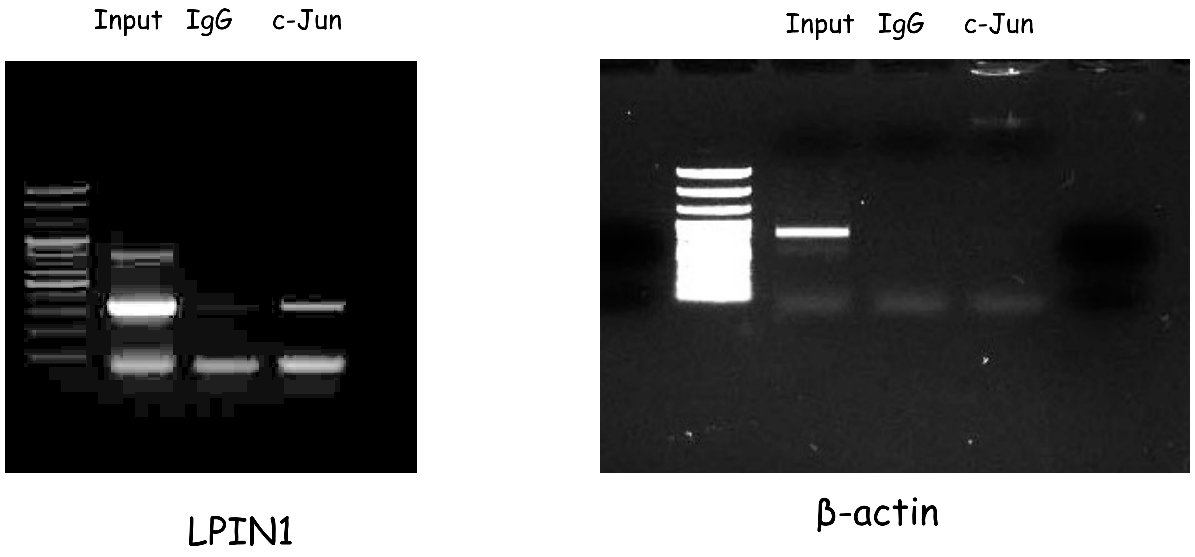

Figure4. F

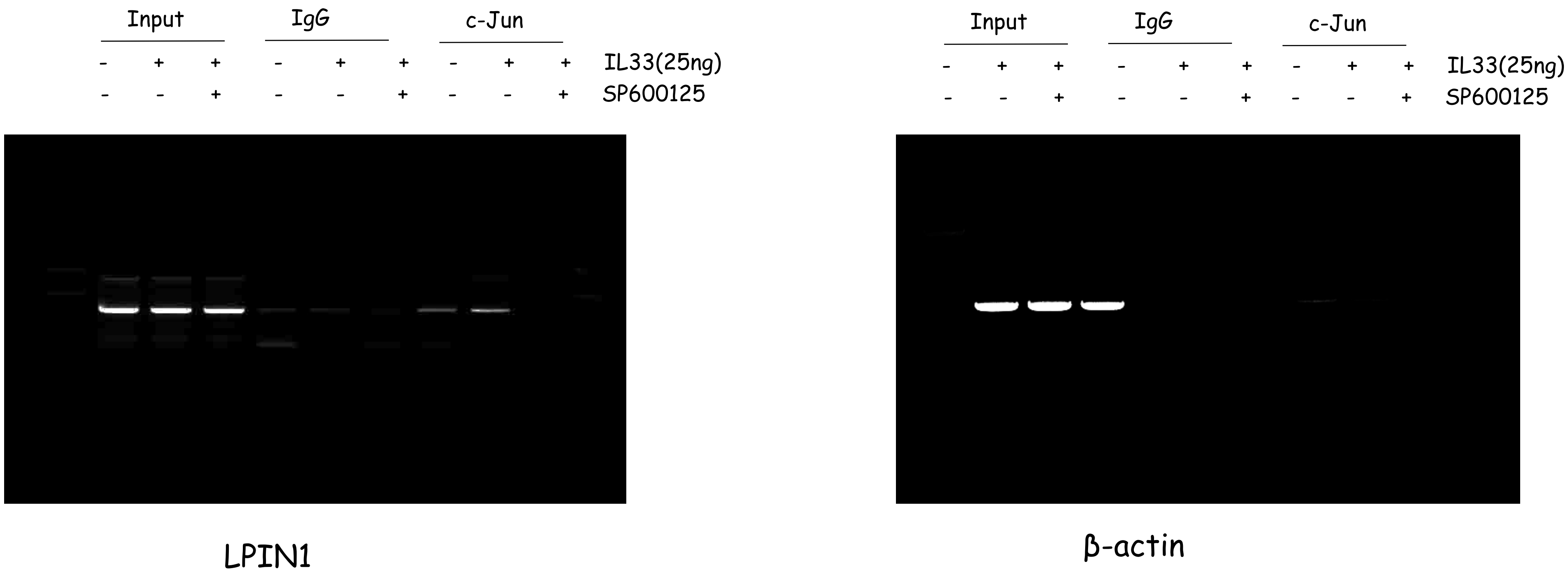

Input
IgG
c-Jun
- + + - + + - + + IL33(25ng)
- - + - - + - - + SP600125
LPIN1
Input
IgG
c-Jun
- + + - + + - + + IL33(25ng)
- - + - - + - - + SP600125
β-actin

Figure6. A

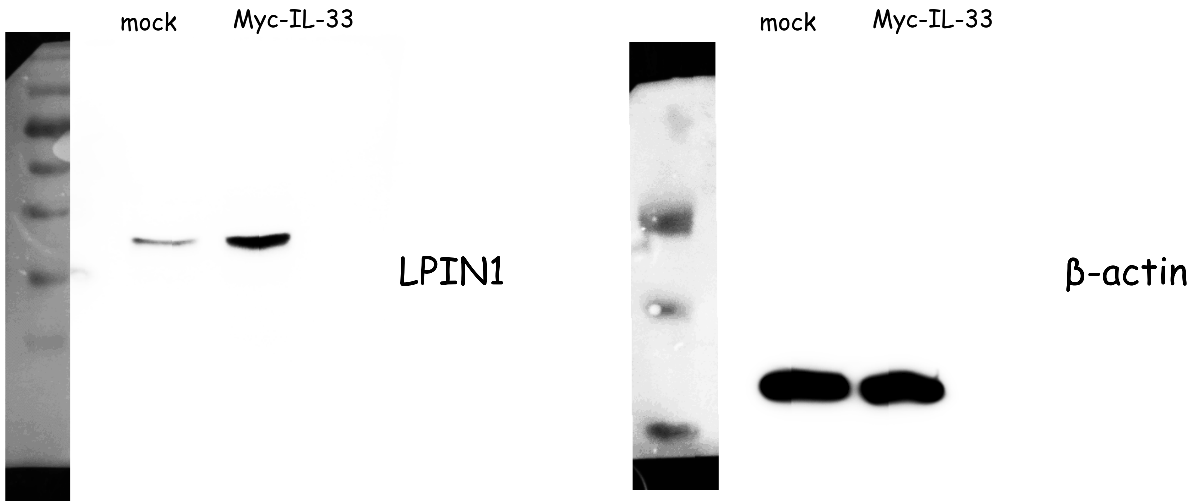

Supplement: Supplementary file 1 [file cancers-13-02174-s001.zip › cancers-1185261-supplementary.pdf]
